# Prolonged Effect of Repetitive Erector Spinae Plane Block in Reducing Thoracic Pain Caused by Lung Cancer

**DOI:** 10.7759/cureus.57130

**Published:** 2024-03-28

**Authors:** Tuba Tanyel Saraçoğlu, Fırat Akbaş, Meryem Onay, Ayten Bilir, Sacit Güleç

**Affiliations:** 1 Department of Pain Management, Başakşehir Çam ve Sakura City Hospital, İstanbul, TUR; 2 Department of Pain Management, Gaziantep City Hospital, Gaziantep, TUR; 3 Department of Anesthesiology and Reanimation, Eskisehir Osmangazi University, Eskişehir, TUR; 4 Department of Pain Management, Eskisehir Osmangazi University, Eskişehir, TUR

**Keywords:** interventional methods, pain treatment, analgesia, cancer pain, lung cancer, thoracic pain, erector spinae plane block

## Abstract

Introduction: Pain significantly affects the quality of life of lung cancer patients. We aimed to evaluate the effect of the erector spinae plane block (ESPB) on pain in these patients.

Methods: We reviewed the medical records of patients with primary lung and bronchial cancer who experienced refractory pain in the thoracic region and underwent repeated ESPBs (three blocks at 24-hour intervals) between 2019 and 2020 in this single-center retrospective study. Visual analog scale (VAS) scores recorded before the procedure and on the first day (first day after the third block) and the first and third months of follow-up in 43 patients were analyzed.

Results: The study population consisted of 31 male and 12 female patients, with a mean age of 56.7 years. The mean pre-procedure VAS score was 8.9±0.8, which showed a significant decrease on the first day (2.9), first month (3.6), and third month (4.6) of the follow-up. Four patients experienced minor complications (pain at the procedure site and hypotension); however, no major complications were observed.

Conclusion: We observed a prolonged effect of repeated ESPBs for ≥3 months. The block efficacy decreased with time; however, an approximately 50% reduction in the VAS score persisted even in the third month. Repetitive ESPBs may be regarded as a straightforward, safe, and replicable intervention to complement medical treatment and diminish the need for opioids in managing lung cancer-related pain.

## Introduction

Lung cancer is the most common cause of cancer-related death worldwide. In 2018, based on GLOBACAN data, there were 2.09 million new cases of lung cancer diagnosed, with 1.76 million fatalities attributed to the disease [1]. Among women, lung cancer remains the third most prevalent cancer and the second leading cause of cancer-related mortality [2].

Lung cancer is usually diagnosed at an advanced stage when symptoms such as pain, fatigue, dyspnea, and coughing appear. Furthermore, patients may develop depression and anxiety in the absence of adequate pain palliation [3].

Pain experienced by patients with lung cancer may be related to treatment or the disease itself. Lung cancer patients frequently experience significant pain, especially during tumor metastasis. Lung cancers frequently metastasize to bone, with up to 39% of individuals with adenocarcinomas suffering from bone metastases. The thoracic and thoracolumbar spine are areas commonly affected by such metastatic spread. The pain from vertebral metastases may initially be tolerable but often escalates rapidly in severity. In advanced stages, patients report persistent, dull bone pain that can be debilitating, accompanied by additional complications such as pathological fractures, spinal cord compression, cachexia, and hypercalcemia, which further exacerbate their discomfort and reduce their quality of life [4]. Visceral pain in the thoracic region may be related to the tumor or lymph node lesions; further, pleural involvement is accompanied by well-localized nociceptive pain [5].

Lung cancer-related pain can be alleviated through medical treatment (administration of paracetamol and nonsteroidal anti-inflammatory drugs, adjuvant treatments, weak opioids, and strong opioids) and interventional treatment methods (epidural catheter ports, spinal pumps, sympathetic blocks, and neurolytic applications) [3].

Among interventional treatment modalities, the erector spinae plane block (ESPB) stands out as an ultrasound-guided fascial plane block employed for managing both acute and chronic pain. This procedure entails injecting a substantial volume of local anesthetic into the fascial plane between the vertebral transverse processes and the erector spinae muscle.

The analgesic effect of ESPB is primarily attributed to the direct diffusion and impact of local anesthetics on neural targets, encompassing dorsal rami branches, spinal nerve roots, ventral rami, and the brachial plexus. Consequently, ESPB offers both somatic and visceral analgesia alongside sympathetic blockade [6].

ESPB is commonly used for postoperative analgesia, particularly in patients undergoing thoracotomy for lung cancer. Nonetheless, existing studies assessing its postoperative analgesic effect have primarily focused on short-term efficacy [7]. Additionally, two cases of continuous ESPB for pain palliation in Pancoast tumors and pleural mesothelioma have been reported [8,9].

To the best of our knowledge, no studies have yet demonstrated the effects of repeated ESPBs in patients with lung cancer. Hence, a more comprehensive understanding of the impact of repeated ESPBs is imperative. Consequently, this study aimed to assess the effect of repeated ESPBs on refractory thoracic pain associated with lung cancer.

## Materials and methods

Study design and population

This study was approved by the Institutional Ethics Committee of the Eskisehir Osmangazi University (No. 25403353-050.99-E.51606) in accordance with the Declaration of Helsinki. We retrospectively reviewed the data of patients with bronchial and lung cancer who were followed up at our hospital's algology clinic for thoracic refractory pain between 2019 and 2020.

The inclusion criteria were as follows: age ≥18 years, diagnosis of primary lung and bronchial cancer from the oncology department, and received recurrent ESPBs due to ineffective medical therapy or inability to tolerate dose escalation.

The exclusion criteria were as follows: had undergone radiotherapy for lung cancer and/or had undergone thoracic surgery prior to interventional treatment and/or within the three-month follow-up period after interventional treatment.

Among the 56 patients who underwent recurrent ESPBs, we excluded eight patients without a sufficient follow-up period, three who underwent radiotherapy for lung tumors, and two who underwent thoracic surgery during the follow-up period. Therefore, we included data from 43 eligible patients.

Intervention

**Figure 1:**
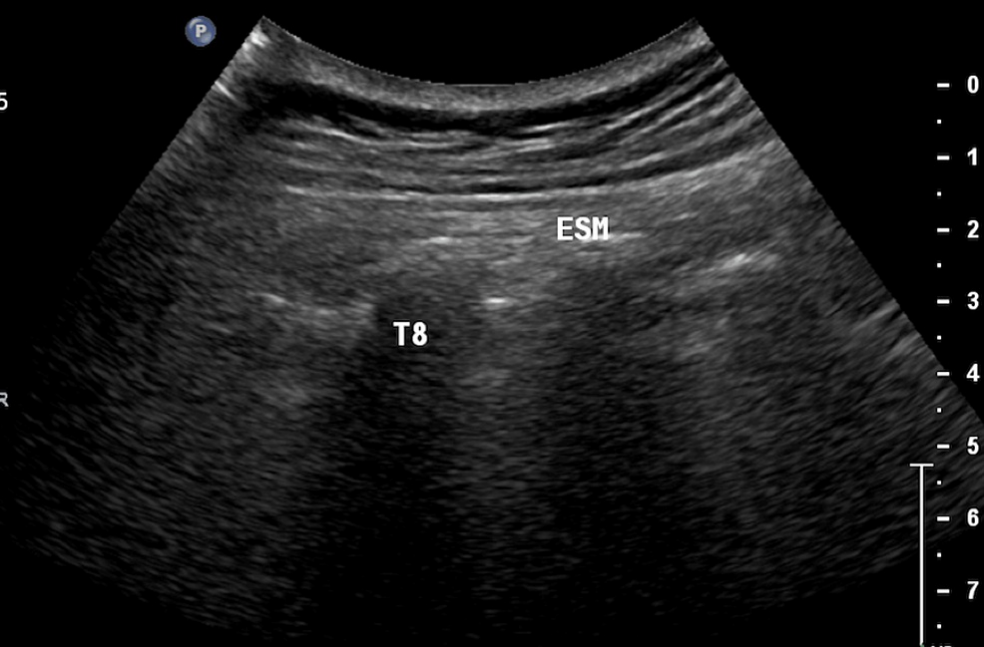
Ultrasound image of the transverse processes and erector spinae muscle. ESM: erector spinae muscle; T8: transverse process of the eighth thoracic vertebra.

**Figure 2:**
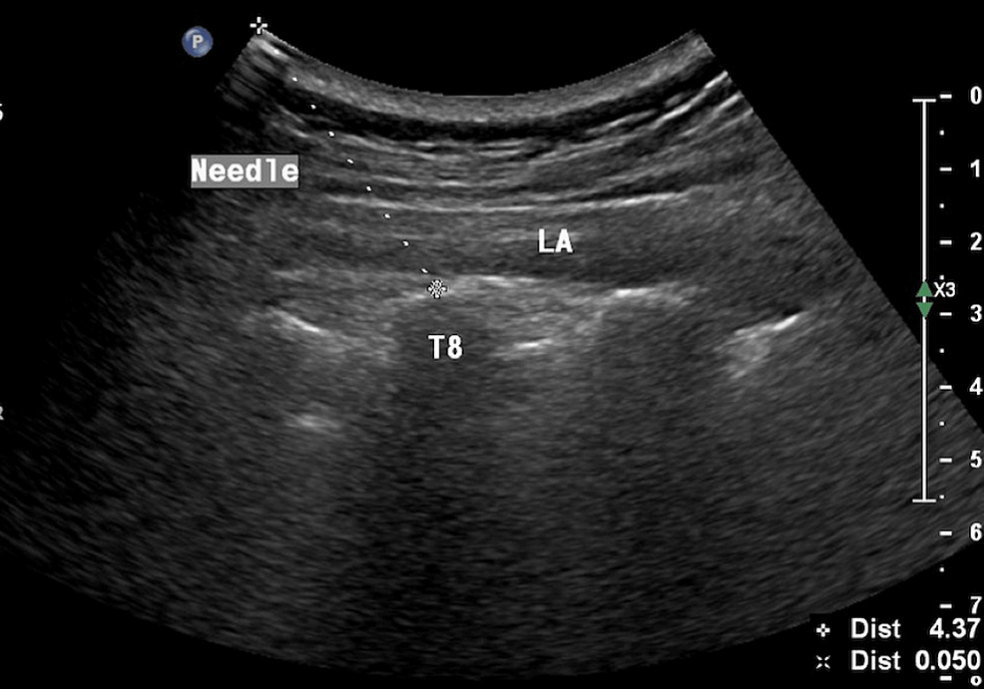
Ultrasound image showing the opening of the plane and spread of local anesthetic LA: local anesthetic.

Before the procedure, the pain intensity of the patients was recorded using a visual analog scale (VAS). After informed consent was obtained, the patients were taken to the procedure room and underwent basic blood pressure, heart rate, and oxygen saturation monitoring. Subsequently, with the patient in the pronated position, the spinous process of the vertebra closest to the region of pain was determined, and localization of the transverse process of the appropriate vertebra was confirmed using a convex ultrasound probe under appropriate aseptic conditions. The probe was sagittally positioned to visualize the rhomboid, trapezius, and erector spinae muscles (Figure 1). Following skin infiltration with 1-2 ml of 2% lidocaine, a 22G 8-cm peripheral block needle was inserted between the erector spinae muscle and the vertebral transverse process using an in-plane approach. After touching the transverse process, the needle location was confirmed through hydrodissection using 1-2 ml saline. After opening the plane, 20 ml of 0.25% bupivacaine was administered (Figure 2) [10]. Following the procedure, blood pressure, heart rate, and oxygen saturation were monitored and recorded at 15-min intervals for 1 h. The sensory block was confirmed using a pinprick test 1 h after ESPB. After the procedure, patients were routinely followed up for 4 h in private patient rooms and monitored for complications.

Follow-up

VAS scores were recorded one day after the third block (first control) as well as in the first and third follow-up months.

Statistical method

Descriptive statistics utilized mean, standard deviation, median, minimum, maximum, frequency, and ratio values. The distribution of variables was determined using the Kolmogorov-Smirnov test. The Wilcoxon test was used to analyze the dependent quantitative data. IBM SPSS Statistics for Windows (version 28.0; IBM Corp., Armonk, NY) was used for all the statistical analyses.

## Results

We included 31 (72.1%) male and 12 (27.9%) female patients, with a mean age of 56.7 years (Table 1). The lung cancer types and stages of the patients are shown in Table 1.

**Table 1:** Patient demographic data and lung cancer types and stages Min: minimum; Max: maximum; Med: median. All values are n (%) except where indicated by an asterisk.

		Mean±SD (Min-Max-Med)
Age		56.7±10.4* (38-74-59)*
Sex	Male	31 (72.1%)
	Female	12 (27.9%)
Lung cancer type	Squamous cell carcinoma	15 (34.8%)
	Small cell carcinoma	8 (18.6%)
	Large cell carcinoma	7 (16.2%)
	Adenocarcinoma	13 (30.2%)
Lung cancer stage	Stage 2	15 (34.8%)
	Stage 3	21 (48.8%)
	Stage 4	7 (16.3%)

During the follow-up period, no alterations were observed in the cancer stages of the patients.

**Figure 3:**
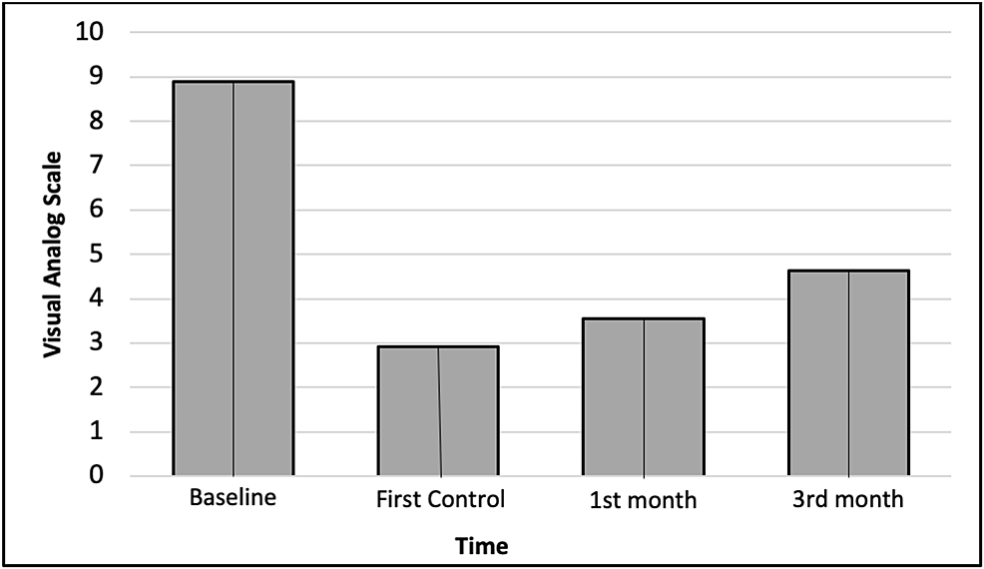
Changes in visual analog scale scores with time

The mean preoperative VAS score was 8.9±0.8, which significantly decreased at the first control (2.9), as well as at the first (3.6) and third (4.6) follow-up months, respectively (all p < 0.001). Furthermore, the mean VAS score significantly increased in the first and third months compared with the first control and first month, respectively (all p < 0.001; Table 2; Figure 3).

**Table 2:** First control, first month, and third month VAS scores of patients w: Wilcoxon test (p < 0.001). *Comparison to baseline; ^‡^Comparison to previous measurement.

	Min	Max	Median	Mean±SD	p-Value*	p-Value^‡^
VAS score						
Baseline	7.0	10.0	9.0	8.9±0.8		
First control	2.0	6.0	3.0	2.9±1.1	<0.001^W^	
Month 1	2.0	6.0	4.0	3.6±0.9	<0.001^W^	<0.001^W^
Month 3	3.0	8.0	4.0	4.6±1.1	<0.001^W^	<0.001^W^

**Figure 4:**
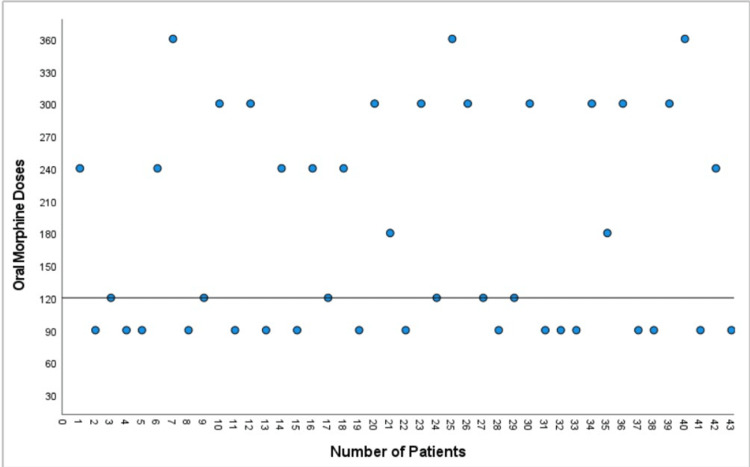
Morphine equivalent oral daily doses used by patients Medication doses are given in mg/day. Median: 120 mg/day.

**Figure 5:**
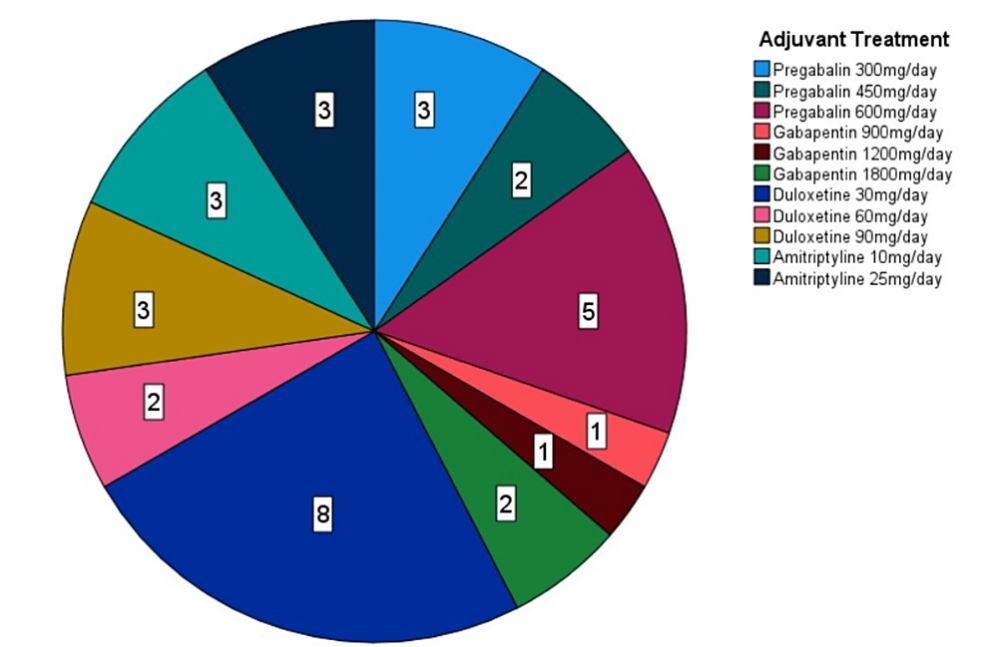
Adjuvant treatments used by patients (n=33)

The opioids used by the patients were converted into the equivalent oral morphine doses, and the daily oral morphine dose received by each patient is shown in the graph (Figure 4). Additionally, the adjuvant treatments used (amitriptyline, duloxetine, gabapentin, pregabalin) and their daily doses are shown in Figure 5.

It was found that patients' daily oral morphine doses and adjuvant treatments did not change during the follow-up period. Four patients experienced minor complications (pain at the procedure site and hypotension); however, no major complications were observed.

## Discussion

The characteristics and duration of lung cancer-related pain are influenced by numerous factors, including the disease itself, treatment, and the psychological state of the patient. Reportedly, 38% of patients experiencing lung cancer-related pain endure discomfort at two or more anatomical sites, with the chest and lumbar spine being the most frequently affected areas [11,12]. Additionally, 13% of patients exhibit pain syndromes associated with thoracic surgery and radiotherapy.

To date, to the best of our knowledge, there exists no entirely safe and effective intervention for lung cancer-related pain. This lack of treatment options can be attributed to the varied etiology of cancer-related pain and the psychological burden of the disease.

Similar to other pain conditions, lung cancer-related pain is primarily managed using pharmacological treatment. While modifications have been made, the stepwise therapy recommended by the World Health Organization remains valid. First-line paracetamol and nonsteroidal anti-inflammatory drugs are generally inadequate when used alone but are crucial components of multimodal analgesia protocols [13]. They are especially indispensable in the presence of bone metastasis. However, gastrointestinal, renal, cardiovascular, and hepatic side effects may pose significant drawbacks, particularly with long-term use, given the frequency of comorbidities in patients with cancer [14].

Opioids have been shown to provide highly effective analgesia to treat cancer-related pain and can be administered alone or in combination with other pharmaceuticals. Opioids may have fewer side effects when administered under analgesic ladder therapy guidance; however, opioid therapy may entail gastrointestinal symptoms such as nausea, vomiting, and constipation, as well as central effects ranging from sedation to unconsciousness and respiratory depression. These risks are particularly notable when considering the overall disease course of cancer and the similar adverse effects associated with chemotherapy and radiotherapy [15]. Adjuvant agents such as pregabalin, gabapentin, and duloxetine can be used to treat patients with cancer-related neuropathic pain or in cases of inadequate current treatment [16]. Despite appropriate medical treatment protocols, adequate analgesia may not be achieved. Interventional therapies may be considered as an alternative treatment or to support medical therapy in cases where medical treatment alone is inadequate or dose increases are not tolerated. However, interventional treatments are considered last-line treatments due to risks such as pneumothoraces, hematomas, and infection. Since the general condition of cancer patients can deteriorate rapidly due to the "analgesic lift" phenomenon during cancer pain treatment, interventional procedures can be applied at any stage after a holistic and personalized evaluation of the patients' general condition, pain severity, potential benefits of the procedure, and potential complications arising from the procedure [17].

Interventional treatments for lung cancer-related thoracic pain include neurolytic sympathetic blocks, intercostal blocks or neurolysis administration, thoracic dorsal root ganglion (DRG) blocks or radiofrequency administration, epidural or spinal catheters, and port pump systems [18-20]. However, the anatomical structure of the medulla spinalis in the thoracic region impedes the administration of DRG and sympathetic blocks.

While neuraxial opioid/local anesthetic administration via a catheter or port/pump offers effective analgesia, it presents several limitations. These include technical challenges in the thoracic region, hypotension, risk of epidural hematoma and infection, and potential respiratory depression. Moreover, neuraxial application cannot be performed in patients with impaired coagulation [21].

Intercostal nerve blocks are relatively easy to administer; however, they are ineffective in countering visceral pain. Intercostal blocks typically require administration at two or more levels. This block has been shown to be particularly effective in thoracic pain such as intercostal neuropathy. It is a safe method when applied with imaging guidance, but patients may experience serious complications such as pneumothorax [22]. Nevertheless, these interventional techniques are commonly employed and can be effective when appropriately chosen.

Administration of the aforementioned treatments is recommended after pharmacological intervention, given their possible complications in the classical doctrine; however, this approach may not be appropriate for all patients. For example, there may be contraindications for interventional methods following pharmacological treatment, including the patient reaching the terminal period and deterioration of the general condition, which may result in uncontrolled pain during the final stages. This is a clinical dilemma, as some interventional methods have better tolerability than high-dose opioids; thus, since localized pain may be controlled using relatively simple and safe interventional methods, it is plausible that interventional methods can also be applied before pharmacological treatments. Conversely, the risk of complications associated with interventional treatments cannot be ignored [13]. It is important to remember that pain management is personal and the choice of treatment should be made on a patient-by-patient basis.

Ultrasonographic imaging, particularly for nerve and field blocks, has facilitated the safe implementation of certain interventional methods and the innovation of new approaches. ESPB administration under ultrasonographic imaging has recently been described.

ESPB is a field block performed by injecting a local anesthetic between the deep fascia of the erector spinae muscle and the transverse process of the vertebra. It was first described by Fererero et al. in 2016 to relieve thoracic postherpetic neuralgia. Following ESPB administration, the local anesthetic diffuses within the erector spinae plane, situated between the anterior aspect of the erector spinae muscle and the posterior aspect of the vertebral transverse process. This action effectively blocks the dorsal and ventral rami of the thoracic or abdominal spinal nerves, consequently providing analgesia by inducing a sensory block over a wide area [10]. Further, the local anesthetic administered in ESPB is thought to enter the thoracic paravertebral area and block the rami communities that transmit sympathetic fibers; accordingly, the sympathetic block can also effectively help control visceral pain [23]. In contrast, sympathetic blocks carry a risk of pneumothoraxes, hematomas, and infection due to their interventional nature. The significant reduction in acute pancreatitis-related pain following ESPB administration further supports the notion that ESPBs provide visceral analgesia [24].

While ESPBs were initially used to treat chronic pain, most subsequent studies have focused on postoperative analgesia. Several reports have indicated that thoracic ESPB provides effective analgesia and reduces the requirement for morphine following breast surgery and thoracotomy [25,26]. ESPB, which may help effectively treat visceral and neuropathic pain through sympathetic blockade, has gained importance for the treatment of postoperative pain.

Administration of ESPB under ultrasonographic guidance significantly reduced the risk of pneumothorax. A single injection can be administered without requiring sedation or analgesia. Given the effectiveness of ESPB against somatic, visceral, and neuropathic pain, it may be an easy, relatively less invasive, safe, and effective method for palliation of cancer pain.

ESPB has been shown to treat chest pain caused by thoracic malignancies effectively. For example, Ramos et al. found that long-term ESPB administration using a catheter was effective in patients with mesothelioma [9]. Moreover, in another case of shoulder pain due to lung cancer, ESPB with 15 ml of 8% phenol yielded pain relief for three weeks [27]. Furthermore, Sirohiva et al. reported pain relief for >1 month after one-time ESPB application in three cases (one and two cases of lumbar and thoracic cancer-related pain, respectively) [28]. 

In previous plane block and nerve block studies, it was found that the effect and duration of repeated blocks were longer [29,30]. Since we have observed that repeated blocks are associated with long-term efficacy, the blocks applied in our clinic are repeated three times. Catheters were not used because of the increased risk of infection (especially in cancer patients) and displacement, and because patients in our clinic are followed on an outpatient basis.

As a result of our retrospective evaluation, we found that there was no change in the patients' opioid doses throughout the follow-up period. This may be due to the fact that opioid treatment with ESPB led to an approximately 60% decrease in the average VAS score in the first month and an approximately 50% benefit in the third month. Increased relief may be achieved via further repetitions of this procedure using a permanent ESPB catheter, which may allow for reductions in opioid use. Since our study was retrospective, we did not evaluate additional scales such as quality of life and sleep quality. The effect of ESPB on opioid dose requirements and additional scales such as quality of life and sleep quality can be better evaluated through randomized prospective studies with a long follow-up period and control group.

No prospective studies or case series have yet been conducted on this topic. Moreover, the sites and agents used for ESPB differed across available case reports. The present study included the largest number of ESPB cases for the treatment of cancer-related thoracic pain. Therefore, we consider our results to be statistically significant.

The limitations of our study include the absence of catheter usage, leading to frequent interventions for patients, the small sample size, the inability to assess scales such as quality of life and quality of sleep, and our reliance on retrospective evaluation. If the patients could be followed up with a catheter and patient-controlled analgesia, potential changes in opioid dose could be observed. In addition, although the patients' cancer stages did not change, we could not share other oncological treatments such as chemotherapy and targeted therapies due to limited data, which is one of the main limitations of our study. Prospective studies with a larger sample size, control groups, and data supported not only by pain scales but also by other scales can better evaluate the potential changes and safety of ESPB in managing thoracic pain associated with lung cancer.

## Conclusions

In conclusion, since ESPB administration provided significant relief for three months and was well tolerated without major complications, it may be considered an option for cancer-related thoracic pain. ESPB may be an easily applied and reproducible method in cases where central blocks cannot be performed to support medical treatment, or the patient cannot tolerate complex interventions. However, large-scale, prospective, and controlled studies are needed to confirm the efficacy and safety of ESPB.
